# Behind the scenes: Impact of virtual backgrounds in educational videos on visual processing and learning outcomes

**DOI:** 10.16910/jemr.16.3.4

**Published:** 2023-10-19

**Authors:** Leen Catrysse, Andrienne Kerckhoffs, Halszka Jarodzka

**Affiliations:** Open Universiteit, Department of Online Learning and Instruction, Heerlen, the Netherlands

**Keywords:** Education, instructional video, multimedia, eye tracking, virtual background, working memory capacity, learning outcomes

## Abstract

The increasing use of instructional videos in educational settings has emphasized the need for a deeper understanding of their design requirements. This study investigates the impact of virtual backgrounds in educational videos on students' visual information processing and learning outcomes. Participants aged 14-17 (N=47) were randomly assigned to one of three conditions: a video with a neutral, authentic, or off-topic background. Their prior knowledge and working memory capacity (WMC) were measured before watching the video, and eye tracking data was collected during the viewing. Learning outcomes and student experiences were assessed after viewing. The eye tracking data revealed that a neutral background was the least distracting, allowing students to pay better attention to relevant parts of the video. Students found the off-topic background most distracting, but the negative effect on learning outcomes was not statistically significant. In contrast to expectations, no positive effect was observed for the authentic background. Furthermore, WMC had a significant impact on visual information processing and learning outcomes. These findings suggest that educators should consider using neutral backgrounds in educational videos, particularly for learners with lower WMC. Consequently, this research underscores the significance of careful design considerations in the creation of instructional videos.

## Introduction

Even though instructional videos are being used since the early 1900s as a means to deliver instruction, research on learning from instructional videos only really started to thrive in the past few years (14; 19). This relatively recent sense of a need for knowledge concerning video design, can be explained by the substantial increased use of instructional videos in formal as well as non-formal learning environments (5; 14; 40). This development has taken an even further flight during the Covid-19 pandemic when schools were closed and educators all over the world had to turn to online education, including recording their own instructional videos. However, guidelines on how to design these instructional videos are limited (14; 19). Since instructional design based on intuition alone, endangers an efficient and effective learning experience (44), it is important to gain more insight into questions related to the design of instructional videos.

As a research area, instructional video design, offers a broad scale of research topics to explore like, how to present the information, the social and emotional effects of videos, or the added value of videos over static images, to name a few (for an overview, see e.g., 5; 14). Findings of studies concerning some of these topics have already led to research-based principles for the design of instructional videos, such as the pacing principle or the signaling principle (19), but there are still a lot more questions that can be asked and answers that need to be found (14).

One of those questions concerns the effect of a video’s background on learning outcomes. Online software, such as Microsoft Teams or Zoom offers the possibility to choose a virtual background. By doing so, one prevents the other interlocutors from seeing the actual setting from which one is working or presenting. There can be several reasons why one would choose to change the background image, for instance privacy, a lack of a suitable work area, or personal preferences. Instructors, like teachers who work from home, might also wish to use this option for their instructional videos or online courses. It would be helpful for them if there were guidelines concerning background options, so they would know whether it matters which background they use, and if so, which sort of background would be best to choose. The chosen background should, at least, not hamper learning, and preferably, even optimize learning.

To our knowledge, the role of an instructional video’s setting or background, has only been explored by Merkt et al. (40). They compared learning outcomes from watching a video that was filmed in front of a white wall, with learning outcomes from watching a video filmed in an authentic setting, which was a greenhouse in their case. They expected to find that the video’s setting would affect the learning outcomes, but it turned out that there were no differences between both conditions (40). So far, instructional video research has neglected the potential importance of virtual backgrounds in home-made instructional videos and how it affects both a learner’s visual information processing and learning outcomes. Therefore, the aim of this study is to investigate whether a virtual background in an instructional video affects visual processing and learning outcomes.

### The role of backgrounds in educational videos

The Cogntive Theory of Multimedia Learning (CTML) explains how people learn from multimedia presentations such as instructional videos (38). The theory is based on three assumptions. The dual-channel assumption (3; Paivio, 1986), which considers that people process incoming textual and pictorial information through two separate channels. The limited-capacity assumption (3; Sweller et al., 2011), which suggests a limit to the amount of information each channel can process at a time. And the active processing assumption (Mayer, 2009; Wittrock, 1989) which states that people should actively attend to useful information in order to transfer this information into the working memory. There, the information should be organized into mental models so it can be connected to prior knowledge already present in the long-term memory (38).

The limited-capacity assumption is also reflected in the Congitive Load Theory (Sweller et al., 1998, 55): To be able to learn, the learner must have enough capacity available in working memory for relevant information processing, while unnecessary processes must be avoided (55). According to the CLT, there are three types of cogntive load that a learner deals with during learning: intrinsic, extraneaous and germane load (Sweller et al., 1998, 55). The amount of intrinsic cognitive load is determined by the complexity of the subject matter itself and the learner’s prior knowledge. This load has nothing to do with the way how the learning material is presented, and it cannot be changed. The amount of extraneous cognitive load on the other hand, is related to the instructional procedures or design. This can be changed, for instance by altering the material’s design. Germane load refers to working memory resources dealing with intrinsic rather than extraneous cognitive load.

Both theories have led to numerous design guidelines (39; 55). The focus of the current study is on the guideline concerning seductive details (20). Seductive details are interesting but irrelevant details, that are not part of the subject matter. They are only added to the learning material to make the subject and material more appealing to the learner, and thus create interest (37). Seductive details might increase extraneous cognitive load and might hamper learning since non-relevant information might take up much needed working-memory capacity. The overall conclusion of an extensive meta-analysis by Rey (49) was that seductive details hamper learning. This could be seen in retention as well as transfer performance (49). According to Merkt et al.’s (40) distraction hypothesis, a video’s background might be distracting to the learner. Adding a background to an instructional video could be considered as seductive details, since it is not integrated in the subject matter that must be learned (40).

Merkt et al. (40) assumed a competing hypothesis when studying the effects of an authentic versus a neutral video background. Their expertise hypothesis assumes that learners are more likely to focus on a teacher who they consider to be an expert, than on a teacher they consider to be a novice (7). Indeed, several studies found that instruction from an expert, or even a perceived expert, led to better learning outcomes (6; 25; 30). In certain situations, learners have no clue about the teachers’ level of expertise. For example, a learner might see an instructional video about a topic the learner has little or no prior knowledge about explained by a teacher that the learner does not know. In this specific scenario, learners rely on observable factors to form an opinion (53), such as clothing styles (41), gender (23), or age ( 25). Next to these personal features, people are also influenced by the context in which they see another person (53). Wittenbrink et al. (60), for instance, showed that the same Black person was perceived differently, if the background picture was a church interior instead of a street corner. The street corner picture led to a larger amount of negative automatic responses (60). Therefore, in line with Merkt et al. (40), we assume that also an instructional video’s background could serve as a sign of expertise. The background might serve as sign of expertise if it represents the usual work environment of the presenter (40). Seeing the instructor in their natural habitat so to speak, could have a positive effect on the perceived expertise, which in turn might have a positive effect on the learning outcomes.

### Working memory capacity

As already stated, leading memory researchers, agree that the capacity of the working memory (WMC) is limited (e.g., 3; 59), but it also varies across individuals (16). In this view, the WMC is not only determined by the limited number of elements one can remember, but rather on the ability to control one’s own attentional resources (16, 17; 54). In this approach, WMC indicates how capable a person is in avoiding distraction, while staying focused on information that should be noticed and retained to be further processed (18; 54).

Research already demonstrated that seductive details are more harmful to learners with low attention control and thus low WMC (49; 50). When videos contain backgrounds including seductive details, they might be more harmful for learners with low WMC since they have difficulties to control their attentional resources.

### Visual information processing

Over the years, eye tracking has proven to be a useful method to gain insight into the visual and cognitive processing of information while learning (2; 12; 26; 31). For instance, Tsai et al. (57) studied the effects of seductive illustrations in a PowerPoint presentation. Their eye tracking data showed that seductive details drew learners’ attention away from the relevant pictures in the presentation. Sanchez and Wiley (50) found that participants with high WMC paid less attention – as indicated by eye tracking data – to seductive images that were added to a Web page.

In instructional videos, several areas of interest (AOIs) can be considered such as the teacher, the PowerPoint slides and the background. The background is considered as a less relevant area compared to the others. A bigger focus of attention on the background, instead of the other areas, could indicate that the background is a distraction comparable to a seductive detail (50) or that a learner pays more attention to the background to form an opinion on the instructor’s expertise.

### Research questions

Like Merkt et al. (40), this study investigates the effect of a video’s background on learning outcomes. But instead of comparing videos filmed in different environments, we compared home-made instructional videos with different virtual backgrounds. Merkt et al. (40) defined an expertise hypothesis and a distraction hypothesis. Their expertise hypothesis states that a learner pays more attention to an expert. Therefore, a virtual background that reflects an authentic work environment of the teacher, might contribute to the perception of this teacher as being an expert, and therefore, could positively affect learning outcomes. Their distraction hypothesis on the other hand, states that an authentic background might cause more distraction as the learner would pay less attention to the relevant subject matter, and thus, hamper learning (40). In the current study we compare such authentic to neutral virtual backgrounds and add a third condition with an off-topic background, which is often used in online meeting software. Additionally, we investigate to which extent a learner’s ability to control their attention (49; 50) might interfere with the overall effect of the backgrounds. To do so, we take learners’ working memory capacity (WMC) into account. Finally, we use eye tracking to explore whether and if so, how, a learner’s visual information processing is affected by these different backgrounds (37).

The first research question examined the effect of background and WMC on visual processing (RQ1). We expect that learners allocate more attention on the backgrounds including (seductive) details (authentic and off-topic background) than the neutral background (RQ1a). Additionally, we expect that individuals with a higher WMC will be less affected by distracting backgrounds (authentic and off-topic) than individuals with a lower WMC and will allocate less attention to distracting backgrounds (RQ1b).

Our second research question examined the effect of the background and WMC on learning outcomes (RQ2). If the expertise hypothesis (40) was correct, then the authentic virtual background would lead to better learning outcomes than the neutral and off-topic condition, since it functioned as a sign of expertise. If the distraction hypothesis was correct, then the neutral virtual background would lead to better learning outcomes, because it contained no seductive details that might distract the viewer. The off-topic condition would make clear if the background mattered at all, since this background contained potentially distracting features that were unrelated to the learning material and it could not provide a sign of expertise (RQ2a). Regarding the influence of WMC on learning, we expect that individuals with a higher WMC will be less affected by distracting backgrounds (authentic and off-topic) than individuals with a lower WMC and their learning outcomes will be unaffected by virtual background whereas individuals with a lower WMC will have weaker learning outcomes in distracting backgrounds (RQ2b).

## Methods

### Design

A between-subjects experimental design was used. Participants were randomly assigned to one of the three conditions: neutral, authentic, or off-topic. The background of the video was the only difference between the conditions, and thus the independent variable. Participants’ prior knowledge as well as their WMC were measured as potential control or moderator variables. The eye tracking and learning outcome measures were the dependent variables.

### Participants

The participants (*N*=54) were students from secondary education and were between 14 and 17 years old (*M*=15.57, *SD*=1.02). Due to common problems with calibration or data quality, we maintained data of 47 participants (12 male and 35 female) for the analysis. All participants spoke and understood Dutch on a native or near-native level, and had normal or otherwise corrected-to-normal vision. Participation was on a voluntary basis. There was no advantage for students who participated, nor was there a disadvantage for students who did not participate.

### Materials

The topic of the instructional video was glaciers and lasted 8 min and 55 s. The instructor showed some examples of glaciers and of landscapes formed by them, she explained what a glacier was, discussed five typical aspects of glaciers, and finally talked about the uncertain future of glaciers. While explaining, she showed fifteen different slides containing either pictures, keywords or schematic images. Some of the slides had an animation, such as arrows that appeared to clarify the position of a typical formation. The slides were not visible during the entire video and there were scenes in which only the instructor was present without slides.

**Figure 1.**
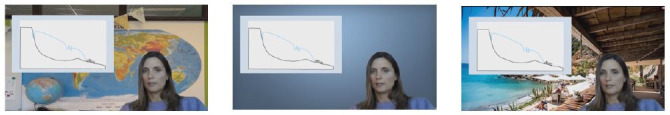
Screenshots of the instructional videos with different virtual backgrounds.

The instructional videos were made by recording a presentation in Zoom. The backgrounds of the videos could be added later, so that there would be three videos which were identical except for the background. The teacher in the three conditions was thus identical and other cues that could be used to derive expertise form (such as age, clothing, and gender) were kept constant. The usual work environment of a geography teacher would be a geography classroom. Therefore, we used a photograph of the geography classroom of the students’ participating in this study as the virtual background for our authentic background (Figure 1, left). For the neutral background, no background was added and a grey background was chosen (Figure 1, middle). For the off-topic background, a background of a beach club was chosen since it has nothing to do with the topic of the video and therefore will also not act as a sign of expertise (Figure 1, right).

Working memory capacity as an individual difference variable, was measured through the Letter-Number Sequencing test. This is a subtest for the measurement of working memory and attention (13) adapted from the Wechsler Adult Intelligence Scale IV. The participant listened to a pre-recorded set of letters and numbers (e.g., K-4-C-2-S) and then repeated these, but not in the same sequence as they were presented. The letters and numbers had to be placed in numerical and alphabetical order (e.g., 2-4-C-K-S). There were ten levels, each level contained three sequences. The amount of numbers and letters in a sequence gradually increased. Level 1 and 2 contain two elements per sequence, Levels 3 to 5 contain three elements per sequence, Level 6 contains four items per sequence, Level 7 contains five items per sequence, Level 8 contains six items per sequence, Level 9 contains seven items per sequence and Level 10 contains eight items per sequence. Participants obtained one point for each correct sequence. If a participant missed on all sequences within a certain level, the test was stopped. The points were summed up to a total score with a minimum of 0 and a maximum of 30 points. We created three WMC groups; high, medium, and low. Participants who made it to Level 7 were placed in the medium group (*N* = 25), those who made it to Level 6 or lower in the low group (*N* = 10), and those who made it to Level 8 or higher in the high group (*N* = 12).

The eye tracking data was recorded by a Tobii Pro Nano eye tracker. The video was presented on a HP ProBook 650 G2 (15,6” monitor, display resolution of 1366 x 768, set 60 to 80 cm in front of the participant) via Tobii Pro Lab (version 1.162.32461 x64) software. The audio was transferred through Tecknet headphones. We used the Tobii I-VT fixation algorithm for fixation identification (45). The maximum time between fixations was set at 60 milliseconds and the maximum angle between fixations at .5 degrees. The eye tracking data used in this research, was based on the viewer’s interaction with the background, the teacher, and the PowerPoint slides. Therefore, we created three areas of interest (AOIs). The background and PowerPoint AOIs were static, they both did not change in shape or position. The AOI background was activated during the entire video, the AOI PowerPoint was only active if a PowerPoint slide was visible. The teacher AOI was a dynamic AOI which means that its shape an position were adapted to the teachers’ movements. This AOI was also active during the entire video. We calculated the total time spent within each AOI. This was the sum of the total duration in milliseconds of all fixations a viewer made within the AOI.

This study measured prior knowledge as a covariate, since differences in prior knowledge might strongly affect the posttest results (e.g., 15; 34; 47). The prior knowledge was assessed through a paper-and-pencil test in Dutch. It consisted of nine glacier-related terms such as Lambert Fisher or firn. These same terms appeared again in the instructional video. Participants were asked to indicate whether they were familiar with the term and to briefly write down what they knew about it. There was a maximum score of 27 points that could be obtained. One point per familiar item and one or two extra points for the explanation. The rating was based on a rubric containing keywords that should be mentioned in the explanation of the item. For instance, if a participant indicated that she or he was familiar with the term Lambert Fisher, this person obtained one point. If she or he mentioned that it is the biggest glacier in the world and that it is situated in Antarctic, this person obtained two extra points. If the participant only mentioned one of those aspects, then only one extra point was attributed. The first twenty tests were rated by two independent persons. Since the ICC score of .97 showed a good reliability (28), the remaining tests were rated by one person. The score of the rater assessing all tests was used for the analysis.

The learning outcomes were also assessed through a paper and pencil test. It consisted of 13 open-ended questions since these might be more sensitive to the differences between the instructional conditions (36). There were 3 transfer questions and 10 retention questions. This research developed a grading rubric containing either the exact answers, or the keywords that needed to appear in the answers in order to obtain points. Participants could obtain a total of 22 points for the test. Fourteen points for the retention questions and 8 points for the transfer questions. An example of a retention question is: Indicate the location of the Lambert Fisher glacier and the Kutiah Lungma glacier. The participant obtained half a point for each, if the answer was correct. An example of a transfer question is: a glacier in Italy has been covered with white cloth, explain why this might have been done. To obtain the maximum of three points the answer should contain the following words, or words similar to: lack of snow, reflection, warming up. Again, the first twenty tests were rated by two independent raters. The ICC score for the total test, as well as for the retention questions, was .98. The ICC score for transfer questions was .97. Because of these high scores, the remaining tests were rated by one person. The score of the rater assessing all tests was used for the analysis.

To shed further light on the outcomes of this research, participants were asked to answer six statements concerning their learning experience. For this they could answer on a scale form 1 (not at all) to 7 (very much). The statements were based on the questions asked in the study of Merkt et al. (40). They concern distraction (“I found the background of the video to be very distracting”), difficulty (“The topic of the video was very difficult”), comprehension (“After watching the video I had a good understanding of different glacier formations”), quality of instruction (“The explanations given in the video were very clear”), and expertise of the instructor (“The teacher was very competent”). The last question is a manipulation check (“There was a good fit between the video and the background”).

### Procedure

Participants were tested in individual sessions, which took about 50 minutes. First, there was a brief welcome during which the procedure was explained. The experimenter verified if the informed consent was signed by the participant, and a parent if applicable. After this the participant answered a few demographic questions concerning age, sex, study year, and level. This was followed by the paper-and-pencil prior knowledge test. Once this was finished, the WMC was assessed. Participants put on the headphones and, after a sound check, the actual test started. The experimenter kept the score. Next, the participant received a brief explanation about the eye tracking procedure, if necessary, participants were asked to remove their eye make-up. The experimenter made sure the distance between the screen and the participant was about 60-70 cm. Then the five-point calibration was started. Once this was validated the participant was friendly reminded not to move too much and was invited to start watching the instructional video. After watching the video, the participant took the pencil-and-paper posttest to assess the learning outcomes. Finally, the participant answered a few subjective questions about the learning experience. After this there was a debriefing, however participants were only informed about the exact research topic once all the data had been collected, since they all attended the same school.

### Data Analysis

Initially data was collected of 54 participants. After screening the eye tracking data, seven participants were excluded from the analyses: Five because of a gaze sample (i.e., ratio of eye movements tracked by the eye tracking device) below 90% and two due to outlier analyses.

The datasets and scripts used for the analysis can be found on OSF (https://osf.io/mgsdh/?view_only=d6d3525248d34ca786d3c9ad8bbcdf20). We used R version 4.3.0 (48) and the following R packages: car v. 3.1.2 (21), emmeans v. 1.8.6 (33), ggpubr v. 0.6.0 (27), here v. 1.0.1 (42), lsr v. 0.5.2 (43), openxlsx v. 4.2.5.2 (52), pgirmess v. 2.0.2 (22), rmarkdown v. 2.22 ( 1; 61; 62), tidyverse v. 2.0.0 ( 58) and ordinal v.2022.11.16 (8. In a first step, descriptive statistics (means and standard deviations) are calculated for prior knowledge, eye tracking measures and posttest measures. In a second step, normality was checked for all variables by inspecting a qqplot and performing a Shapiro-Wilk test for normality. Prior knowledge was not normally distributed and therefore a Kruskal-Wallis test is carried out to examine the relation between background and these measures. Self-report measures were analyzed with ordinal logistic regression. The total fixation duration on the background was also not normally distributed and was logarithmically transformed for further analysis.

Regarding the first research question examining the effect of background and WMC on visual processing, three separate regression analyses were carried out for the three dependent variables: total fixation duration on background, total fixation duration on PowerPoint and total fixation duration on teacher. The independent variables were background (categorical with three factors) and WMC (categorical with three factors) and their interaction effect. For the second research question, examining the effect of background and WMC on learning outcomes, we took the same approach. Three separate regression analyses were carried out for the dependent variables posttest total, retention and transfer. The independent variables are background (categorical with three factors) and WMC (categorical with three factors) and their interaction effect. For both, research question one and two, multiple comparisons of means for levels of WMC within background were carried out.

## Results

Table 1 provides the descriptive statistics of the numeric variables and Table 2 of the ordinal variables. Due to a violation of normal distribution, the prior knowledge measure was tested through a Kruskal-Wallis test. The overall score on prior knowledge was very low compared to the maximum score of 27 that could be obtained, furthermore the Kruskal-Wallis test indicated no significant difference between groups; H(2) = 2.02, p = .363, therefore prior knowledge was not included as a covariate in our analyses.

**Table 1.** Means (M), Standard Deviations (SD), Minimum and Maximum of the Numeric Variables per Condition.

	Authentic (*N*=13)			Neutral (*N*=17)			Off-topic (*N*=17)		
	*M (SD)*	min	max	*M (SD)*	min	max	*M (SD)*	min	max
Prior knowledge	2.69 (1.32)	1.00	5.00	2.00 (1.06)	0	4.00	2.41 (1.46)	0	6.00
Total posttest	10.89 (2.97)	5.50	15.50	10.18 (2.26)	6.50	15.00	9.32 (3.86)	3.00	17.00
Total retention	7.35 (1.90)	5.00	10.50	6.77 (1.26)	4.50	9.00	6.32 (2.51)	2.00	11.00
Total transfer	3.54 (1.31)	.50	5.00	3.41 (1.52)	.50	6.00	3.00 (1.72)	0	6.00
Total time on AOI background in seconds	25.62 (11.61)	7.56	50.64	22.06 (15.51)	2.54	53.56	31.93 (21.54)	3.33	83.92
Total time on AOI PowerPoint in seconds	174.18 (39.79)	114.47	244.32	179.16 (23.38)	131.71	210.46	179.31 (33.42)	115.32	223.83
Total time on AOI teacher in seconds	287.34 (32.00)	235.66	328.91	268.31 (35.33)	206.99	338.27	264.76 (54.60)	166.54	365.28

**Table 2.** Medians of the Ordinal Variables per Condition.

	Authentic (*N*=13)	Neutral (*N*=17)	Off-Topic (*N*=17)
How distracting was the background	3	3	4
How difficult was the topic	3	4	3
Understanding of the topic	5	5	4
How clear were the explanations	5	5	5
How competent was the teacher	5	5	4
How fitting was the background	3	5	1

Ordinal regressions demonstrated that there was a statistically significant difference in the perceived distraction caused by the background across the three different background version groups. Pairwise comparisons demonstrated that the off-topic background group reported a significantly higher perceived distraction than the neutral group (*p* = .019). There was also a statistically significant difference in the perceived fittingness of the background across the three groups. Pairwise comparisons showed that the off-topic background group reported a significantly lower background fittingness than the authentic (*p* < .001) and neutral group (*p* < .001). The authentic background group also reported a significantly lower background fittingness than the neutral background group (*p* < .029). There were no statistically significant differences across the three groups regarding the other measures; difficulty of the topic, understanding of the topic, clear explanations and competency of the teacher.

**Figure 2.**
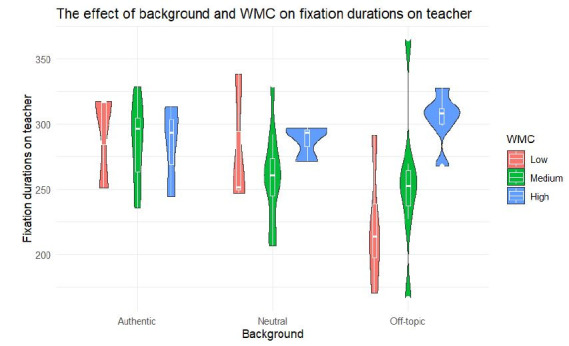
The interaction effect of background and WMC on total fixation duration on the teacher.

To examine the effect of the video’s background and WMC on visual processing (RQ1), three separate regression analyses were carried out for the total fixation duration on the background, the total fixation duration on the PowerPoint and the total fixation duration on the teacher (Table 3). For the background, the adjusted R^2^ is negative and shows a neglectable effect (11) of background and WMC on total fixation duration on the background. No main effect of background and WMC nor an interaction effect was found. Similar results are found for total fixation duration on the PowerPoint, 1.82 % of the variance in total fixation duration can be explained by background and WMC, which is a small effect (11). No main effect nor interaction effect of background and WMC can be found. For total fixation duration on the teacher in the video, 12.77 % of the variance is explained by background and WMC, which is a medium effect (11). An interaction effect was found between background and WMC (Figure 2). More specifically, students with a high WMC spend more attention on the teacher than students with a low WMC for the video with an off-topic background (*p* = .009, Table 4).

**Figure 3.**
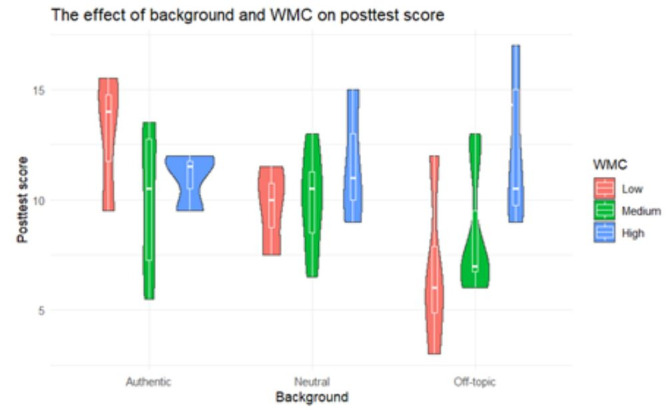
The interaction effect of background and WMC on the total posttest score.

To investigate the effect of the video’s background and WMC on the learning outcomes (RQ2), a regression analysis was conducted for the total posttest score, followed by a regression analysis for the scores on retention and transfer separately. Results of these analyses are displayed in Table 5 and Table 6. Regarding the total posttest score, results demonstrate that 13.76 % of the variance in posttest score can be explained by the background and WMC, which is a medium effect (11). Results demonstrate that the effect of background is depending on WMC. For an off-topic background, students with a high WMC gain a better score than students with a low WMC on the posttest (*p* = .016, Table 6). For the other backgrounds, no significant differences were found (Figure 3).

For the retention questions, similar results were found. 15.09 % of the variance in retention can be explained by the background of the video and WMC, which is a large effect (11). Again an interaction effect was found between WMC and background. For the off-topic background results show that students with a low WMC score lower than students with a high WMC on retention (*p* = .037, Table 6). For the transfer questions, 8.42 % of the variance in transfer can be explained by background and WMC, which is a medium effect (11). Again an interaction effect was found: students with a low WMC gained a lower score on the transfer questions than students with a high WMC for the off-topic background (*p* = .038, Table 6).

**Table 3.** Parameter Estimates of the Regression Analysis for Total Fixation Duration on Background, PPT and Teacher.

	Total fixation duration background (log transformed)				Total fixation duration PPT				Total fixation duration teacher			
	*β*	*SE*	*t*	*pr(>|t|)*	*β*	*SE*	*t*	*pr(>|t|)*	*β*	*SE*	*t*	*pr(>|t|)*
*Estimates*												
Intercept	2.86	.45	6.42	<.001	170.93	18.06	9.46	<.001	295.51	23.06	12.81	<.001
WMC_medium	.29	.53	.55	.587	-3.05	21.59	-.14	.883	-10.19	27.57	-.37	.714
WMC_high	.50	.63	.79	.433	21.20	25.55	.83	.411	-11.62	32.62	-.36	.724
Neutral	.18	.63	.29	.774	15.73	25.55	.62	.542	-16.52	32.62	-.51	.616
Off-topic	.63	.59	1.07	.292	30.43	23.90	1.27	.211	-72.97	30.51	-2.39	.022
WMC_medium*neutral	-.64	.73	-.87	.388	-2.74	29.69	-.09	.927	-8.64	37.90	-.23	.821
WMC_high*neutral	-.52	.89	-.58	.564	-42.42	36.13	-1.17	.248	20.11	46.13	.44	.665
WMC_medium* off-topic	-.38	.72	-.53	.603	-9.87	29.17	-.34	.737	43.04	37.24	1.16	.255
WMC_high* off-topic	-1.24	.80	-1.54	.132	-68.60	32.56	-2.11	.042	92.89	41.58	2.23	.031
*Model fit*												
Adjusted R^2^ (%)	-2.75				1.82				12.77			

**Table 4.** Parameter Estimates of the Multiple Comparisons of Means for Levels of WMC within Background for Attention Allocation (Post-Hoc Tukey Contrast).

	Total fixation duration background (log transformed)				Total fixation duration PPT				Total fixation duration teacher			
	*β*	*SE*	*t*	*pr(>|t|)*	*β*	*SE*	*t*	*pr(>|t|)*	*β*	*SE*	*t*	*pr(>|t|)*
**Authentic background**												
Low WMC – Medium WMC	-.29	.53	-.55	.848	3.05	21.6	.14	.989	10.19	27.6	.37	.928
Low WMC – High WMC	-.50	.63	-79	.710	-21.20	25.5	-.83	.687	11.62	32.6	.36	.933
Medium WMC – High WMC	-.21	.53	-.39	.920	-24.25	21.6	-1.12	.506	1.43	27.6	.05	.999
**Neutral background**												
Low WMC – Medium WMC	.35	.50	.69	.771	5.79	20.4	.28	.957	18.82	26.0	.72	.751
Low WMC – High WMC	.02	.63	.03	.999	21.22	25.5	.83	.687	-8.49	32.6	-.26	.963
Medium WMC – High WMC	-.33	.50	-.65	.792	15.43	20.4	.76	.731	-27.31	26.0	-1.05	.551
**Off-topic background**												
Low WMC – Medium WMC	.09	.48	.18	.983	12.92	19.6	.66	.788	-32.86	25.0	-1.31	.397
Low WMC – High WMC	.74	.50	1.48	.312	47.40	20.2	2.35	.061	-81.27	25.8	-3.15	.009
Medium WMC – High WMC	.65	.43	1.52	.294	34.47	17.4	1.98	.131	-48.42	22.2	-2.18	.088

**Table 5.** Parameter Estimates of the Regression Analysis for Posttest total, Posttest Retention and Posttest Transfer.

	Posttest total				Posttest retention				Posttest transfer			
	*β*	*SE*	*t*	*pr(>|t|)*	*β*	*SE*	*t*	*pr(>|t|)*	*β*	*SE*	*t*	*pr(>|t|)*
*Estimates*												
Intercept	13.00	1.67	7.80	<.001	9.00	1.05	8.61	<.001	4.00	.85	4.73	<.001
WMC_medium	-3.07	1.99	-1.54	.132	-2.07	1.25	-1.66	.105	-1.00	1.01	-.99	.329
WMC_high	-2.00	2.36	-.85	.402	-2.33	1.48	-1.58	.123	.33	1.20	.28	.782
Neutral	-3.33	2.36	-1.41	.166	-3.50	1.48	-2.37	.023	.17	1.20	.14	.890
Off-topic	-6.25	2.21	-2.83	.007	-4.00	1.38	-2.89	.006	-2.25	1.12	-2.01	.052
WMC_medium*neutral	3.31	2.74	1.21	.234	3.44	1.72	2.00	.053	-.12	1.39	-.09	.931
WMC_high*neutral	4.00	3.34	1.20	.238	4.50	2.09	2.15	.038	-.50	1.69	-.30	.769
WMC_medium*off-topic	4.68	2.69	1.74	.090	2.71	1.69	1.61	.116	1.96	1.37	1.44	.159
WMC_high* off-topic	7.42	3.00	2.47	.018	5.33	1.88	2.83	.007	2.08	1.53	1.37	.180
*Model fit*												
Adjusted R^2^ (%)	13.76				15.09				8.42			

**Table 6.** Parameter Estimates of the Multiple Comparisons of Means for Levels of WMC within Background (Post-Hoc Tukey Contrast).

	Posttest total				Posttest retention				Posttest transfer			
	*β*	*SE*	*t*	*pr(>|t|)*	*β*	*SE*	*t*	*pr(>|t|)*	*β*	*SE*	*t*	*pr(>|t|)*
**Authentic background**												
Low WMC – Medium WMC	3.07	1.99	1.54	.284	2.07	1.25	1.66	.234	1.00	1.01	.99	.588
Low WMC – High WMC	2.00	2.36	.85	.676	2.33	1.48	1.58	.267	-.33	1.20	-.28	.958
Medium WMC – High WMC	-1.07	1.99	-.54	.853	.26	1.25	.21	.976	-1.33	1.01	-1.32	.394
**Neutral background**												
Low WMC – Medium WMC	-.24	1.88	-.13	.991	-1.36	1.18	-1.16	.486	1.12	.95	1.18	.475
Low WMC – High WMC	-2.00	2.36	-.85	.676	-2.17	1.48	-1.47	.318	.17	1.20	.14	.989
Medium WMC – High WMC	-1.76	1.88	-.93	.622	-.80	1.18	-.68	.776	-.96	.95	-1.00	.581
**Off-topic background**												
Low WMC – Medium WMC	-1.61	1.81	-.89	.651	-.64	1.13	-.57	.839	-.96	.92	-1.05	.551
Low WMC – High WMC	-5.42	1.86	-2.91	.016	-3.00	1.17	-2.57	.037	-2.42	.95	-2.55	.038
Medium WMC – High WMC	-3.81	1.61	-2.37	.058	-2.36	1.01	-2.34	.062	-1.45	.82	-1.78	.189

## Discussion

The primary goal of this study was to investigate the effects of a video’s background on visual processing and learning outcomes. We compared three conditions; an authentic background, which could have a positive impact on learning since it might be a sign of expertise, a neutral background, which could lead to better learning results because of the lack of potentially distracting seductive details, and an off-topic background which contained seductive details and lacked any sign of expertise, therefore being the background that would negatively impact learning the most, provided that the background had any impact.

Regarding the first research question, we examined the effect of background and WMC on visual processing. Our results demonstrated an interaction whereby in the video with an off-topic background students with a high WMC focused more on the teacher than students with a low WMC. The off-topic background can be considered as a seductive detail. Our results are in line with similar research on seductive images in webpages (50). They found that participants with a high WMC paid less attention to seductive images. Hence, in our study, students with a high WMC are better able at focusing their attention on the teacher, providing important cues through mimics, and hereby neglecting seductive information compared to students with a low WMC.

Concerning the second research question, our results showed that for the off-topic background students with a high WMC performed better at the posttest, for both retention and transfer questions, compared to students with a low WMC. It demonstrates that students with a high WMC are less affected by the potential negative effect of a distracting background. This is in line with several studies on learning from illustrated text in which it was demonstrated that a higher WMC positively affected learning outcomes when seductive details were added (4; 49; 50).

### Limitations of This Study and Suggestions for Future Research

A first limitation of this study is that the entire PowerPoint slides were labeled as areas of interest. For future research, it would be interesting to identify specific relevant areas within the PowerPoint in order to examine whether students did look at the specific relevant parts of the PowerPoint slides and how quickly their attention was focused on relevant parts. In addition, it would be interesting to investigate how attention allocation on the background varies over time. In line with the study of van Wermeskerken et al. (2018), the video could be split into shorter segments to examine whether differences are only present at the start or persist over time.

A second limitation of this study is that the teacher in the instructional video was not making use of explicit gaze and gesture cues. The teacher did use mimics, and we know that natural, non-verbal behavior of a teacher, including mimics, is relevant to understand and learn from him or her (e.g., 51; Zeki, 2019). It is known that faces attract attention and face perception is a highly developed skill (Haxby et al., 2020). It might even be linked to cultural backgrounds because looking at the face is linked to showing interest in the speaker (Haxby et al., 2020). Prior eye tracking research suggests that people from Western cultures tend to look at objects in the foreground, while people from Eastern cultures tend to look more at the background (e.g., 10). It would be, hence, very interesting to see whether such an effect amplifies our findings of the various backgrounds on students’ visual processing and learning outcomes. For future research, we suggest to examine videos in which teachers make use of gaze guidance, gesture guidance or a combination of both (e.g., 46). Research already demonstrated that these cues might guide the attention away from the speaker.

Another suggestion for future research is to investigate the role of expertise of the teacher more explicitly, since, as previously mentioned, the way the viewer experiences the teacher’s expertise, has an impact on learning outcomes (6; 25; 30). Therefore, we suggest to manipulate other observable factors linked to expertise such as clothing styles, gender or age. In addition, it would be interesting to include students with prior knowledge on the topic as well, since this might also impact the way they judge the expertise of the teacher in the video.

Regarding students’ characteristics, this study measured WMC through the Letter-Number Sequencing test, which is based on auditory input, but this could also be measured through other instruments, such as a visual arrays task (35) which is based on visual input. Also, unlike Merkt et al. (40), we did not measure other individual differences such as the participants’ interest in the topic, or their motivation, even though these can impact learning (e.g., 29). Another point is that we measured students’ ratings of the video after they finished the knowledge test, which might have impacted the way they rated the videos.

Concerning the learning environment, Choi et al. (9) suggest that the physical environment in which learning takes place could have an effect on the experienced cognitive load. This research was conducted in a small office. The only two persons present were the researcher and the participant. There were no distractions. In reality learners are likely to watch instructional video’s in a potentially more distraction environment such as a classroom, or their own living rooms or bedrooms. Since this might increase the experienced cognitive load, further research would be needed to investigate whether this amplifies the effects of a video’s background and WMC.

The last limitation and suggestion concerns the choice of the backgrounds. It would be interesting to investigate whether the off-topic background as a whole was distracting, or only certain features in the image. For instance, research has shown that peoples gaze is drawn towards faces (e.g., 32; 56) and there was a women present in the picture used as off-topic background. Our findings might have been different if there was no human present in the off-topic background.

### Conclusions

Even though further research is still needed, this study tends to confirm the distraction hypothesis especially for students with a low WMC. Educators should be aware that their choice for a background in an instructional video, and possibly also during an online course, has an effect on their students’ information processing and learning. It is important that educators are aware of the fact that a distracting background affects students with a low WMC more than those with a high WMC. Because of their visual information processing, students with high WMC are not as easily overloaded compared to persons with a low WMC, giving them a double advantage, the in itself higher capacity and the lower load that needs to be processed. A distracting background could therefore amplify existing differences between students.

### Ethics and Conflict of Interest

The authors declare that the contents of the article are in agreement with the ethics described in http://biblio.unibe.ch/portale/elibrary/BOP/jemr/ethics.html and that there is no conflict of interest regarding the publication of this paper. This research was ethically approved by The Research Ethics Committee of the Open University (U202103165).
